# *CISH* promoter polymorphism effects on T cell cytokine receptor signaling and type 1 diabetes susceptibility

**DOI:** 10.1186/s40348-018-0080-7

**Published:** 2018-02-06

**Authors:** Julia Seyfarth, Heinz Ahlert, Joachim Rosenbauer, Christina Baechle, Michael Roden, Reinhard W. Holl, Ertan Mayatepek, Thomas Meissner, Marc Jacobsen

**Affiliations:** 1 Department of General Pediatrics, Neonatology, and Pediatric Cardiology, University Children’s Hospital, Moorenstr. 5, 40225 Duesseldorf, Germany; 2 German Center for Diabetes Research (DZD), 85764 Munich, Neuherberg Germany; 3 Institute for Biometrics and Epidemiology, German Diabetes Center, Leibniz Center for Diabetes Research at Heinrich Heine University Düsseldorf, 40225 Duesseldorf, Germany; 4 Division of Endocrinology and Diabetology, Medical Faculty, Heinrich Heine University Düsseldorf, 40225 Düsseldorf, Germany; 5 Institute for Clinical Diabetology, German Diabetes Center, Leibniz Center at Heinrich-Heine University Düsseldorf, 40225 Düsseldorf, Germany; 6 ZIBMT, Institute of Epidemiology and Medical Biometry, University of Ulm, 89081 Ulm, Germany

**Keywords:** CIS, *CISH*, Single nucleotide polymorphisms, IL-2, Regulatory T cells, Effector T cells

## Abstract

**Background:**

Impaired regulatory T cell immunity plays a central role in the development of type 1 diabetes (T1D). Interleukin-2 receptor (IL-2R) signaling is essential for regulatory T cells (T_REG_), and cytokine-inducible SH2-containing protein (CIS) regulates IL-2R signaling as a feedback inhibitor. Previous studies identified association of *CISH* promoter region single nucleotide polymorphisms (SNPs) with susceptibility to infectious diseases.

**Methods:**

Here we analyzed allele frequencies of three *CISH* SNPs (i.e., rs809451, rs414171, rs2239751) in a study of T1D patients (*n* = 260, onset age < 5 years, duration > 10 years). Minor allele frequencies were compared to a control cohort of the 1000 Genomes Project. Assigned haplotypes were determined for effects on T1D manifestation and severity. Finally, the *CISH* haplotype influence on cytokine signaling and function was explored in T cells from healthy donors.

**Results:**

We detected similar minor allele frequencies between T1D patients and the control cohort. T1D onset age, residual serum C-peptide level, and insulin requirement were comparable between different haplotypes. Only minor differences between the haplotypes were found for in vitro cytokine (i.e., IL-2, IL-7)-induced CIS mRNA expression. STAT5 phosphorylation was induced by IL-2 or IL-7, but no differences were found between the haplotypes. T_REG_ purified from healthy donors with the two most common haplotypes showed similar capacity to inhibit heterologous effector T cells.

**Conclusions:**

This study provides no evidence for an association of *CISH* promoter SNPs with susceptibility to T1D or severity of disease. In contrast to previous studies, no influence of different haplotypes on CIS mRNA expression or T cell-mediated functions was found.

**Electronic supplementary material:**

The online version of this article (10.1186/s40348-018-0080-7) contains supplementary material, which is available to authorized users.

## Background

Type 1 diabetes (T1D) is a chronic autoimmune disease characterized by the destruction of pancreatic ß cells. Autoreactive effector T cells are involved in T1D pathogenesis, and impaired regulatory T cell (T_REG_) functions promote self-reactive effector T cells [1, 2]. T_REG_ crucially depend on IL-2, and IL-2 receptor (IL-2R) variants are associated with increased susceptibility to T1D [3]. T1D-associated IL-2R variants affect T_REG_ phenotype and function [4]. Therefore, decreased IL-2R signaling is assumed to be central for impaired T_REG_ function in T1D pathogenesis [5]. IL-2R expression and signaling of T cells are tightly regulated, and cytokine-inducible SH2-containing protein (CIS), a member of the suppressor of cytokine signaling (SOCS) family, contributes as a feedback inhibitor [6]. The CIS protein (encoded by the *CISH* gene) plays a role in T cell activation and cytokine-induced proliferation [7]. Several cytokines (including IL-2 and IL-7) induce CIS expression during receptor binding mediated by the Jak/STAT pathway [8]. CIS expression is induced within few hours and inhibits IL-2R signaling by binding of the IL-2Rβ chain and blocking of STAT5 phosphorylation [9, 10].

Previous studies demonstrated important roles of CIS on T cell function during allergic, malignant, and infectious diseases [11–13]. These studies showed that CIS regulated T cell activation and polarization of CD4^+^ and CD8^+^ T cells [12, 13]. Furthermore, genetic *CISH* variants were found to be associated with susceptibility to infectious diseases including tuberculosis [11, 14, 15]. Tuberculosis-associated single nucleotide polymorphisms (SNPs) (i.e., rs809451, rs414171, rs2239751) are located in *CISH* promoter regions and were accompanied with differential CIS expression [11, 16]. Tuberculosis risk allele (i.e., rs414171T/rs809451C) carriers had decreased CIS mRNA expression after IL-2 in vitro stimulation and increased IL-10 serum levels [16]. This suggested increased T_REG_ function of tuberculosis risk allele carriers due to decreased CIS expression [16].

In the present study, we addressed the question if *CISH* SNPs are associated with susceptibility to develop T1D and/or with age at onset and disease severity. We determined minor allele frequencies (MAFs) of three *CISH* SNPs in patients with early-onset and long-term T1D and compared results with controls from the 1000 Genomes database (www.internationalgenome.org) [17]. In addition, we characterized the effects of *CISH* SNPs on cytokine-induced T cell functions.

## Methods

### Patients and healthy controls

Two hundred sixty patients with T1D (mean age 16.4 years, range 10.7–20.9 years; onset age < 5 years, > 10 years diabetes duration) were recruited for the pediatric diabetes biobank within the German Center for Diabetes Research (DZD). Further information on selection and characteristics of the study cohort has been given previously [18]. Healthy adult donors were recruited from the staff working at the University Hospital Duesseldorf (*n* = 14).

### Genotyping of *CISH* SNPs

**Fig. 1:**
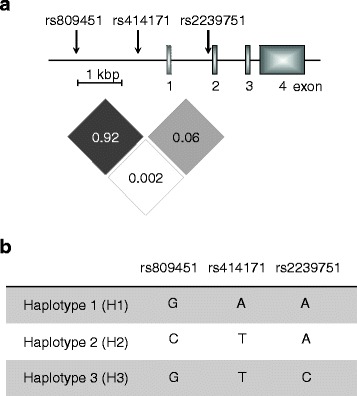
CISH gene region map and haplotypes. **a** Schematic depiction of the *CISH* gene and SNP localization within the promoter region (chromosome 3). Dependency in inheritance of SNPs is indicated as linkage disequilibrium coefficients (*R*^2^, estimated according to https://analysistools.nci.nih.gov/LDlink). **b** Three respective haplotypes were assigned (H1/H2/H3)

DNA was isolated from peripheral blood using QIAamp DNA Mini Kit (Qiagen). TaqMan assays (Applied Biosystems) were used for genotyping of three *CISH* SNPs (i.e., rs809451, rs414171, rs2239751). *CISH* SNP inheritance was analyzed, and linkage disequilibrium was estimated using a publically available tool (https://analysistools.nci.nih.gov/LDlink). The structure of the *CISH* gene region and SNP linkage are depicted in Fig. 1a. Minor allele frequencies (MAFs) were calculated and compared with the European (EUR) cohort from the 1000 Genomes Project [17]. *CISH* SNP variants were assigned to three haplotypes based on previous reports [14, 16]. Because of the predefined number of available biobank samples, we performed post hoc power calculations and included confidence intervals for MAF differences (according to [19]) (Additional file 1: Figure S1 and Table 1).

**Table 1:** *CISH* SNP genotype frequencies of T1D patients and controls

SNP ID	Genotype	Frequencies	MAF T1D	MAF control group^a^	Observed difference (95% CI)	*p* value
rs414171	T/T	2 (0.8%)	12.1%	11.6%	0.49% (−2.82; 4.08)	0.81
	T/A	59 (22.7%)				
	A/A	199 (76.5%)				
rs809451	C/C	2 (0.8%)	11.3%	11.0%	0.31% (−2.90; 3.82)	0.86
	C/G	55 (21.2%)				
	G/G	203 (78.1%)				
rs2239751	C/C	0 (0.0%)	0.8%	0.6%	0.17% (−0.67; 1.41)	0.74
	C/A	4 (1.5%)				
	A/A	256 (98.5%)				

^a^According to 1000 Genomes database (EUR cohort), *n* = 503; 260 T1D patients were included

### Parameters of T1D severity

Residual random serum C-peptide levels and daily insulin requirement were used as proxies for disease severity. C-peptide was measured with a high-sensitivity assay [18]; data on insulin requirement were retrospectively obtained from the German/Austrian nationwide DPV registry [18].

### In vitro T cell assays

For quantification of cytokine-induced CIS mRNA expression and STAT5 phosphorylation, peripheral blood mononuclear cells (PBMCs) from healthy donors genotyped for *CISH* SNPs rs809451, rs414171, and rs2239751 were isolated and stored in liquid nitrogen. Samples were then concomitantly thawed for in vitro assays. For measurement of CIS mRNA expression, PBMCs were stimulated with IL-2 (100 IU/ml, Sigma-Aldrich) or IL-7 (10 ng/ml, Sigma-Aldrich) for 1 or 2 h. mRNA was then isolated and reverse transcribed. CIS mRNA was quantified by real-time quantitative (q)PCR as described previously [20]. A non-stimulated PBMC sample was used to determine ex vivo CIS expression in PBMC. GAPDH was used as a qPCR house-keeping control [20].

For STAT5 phosphorylation, PBMCs were stimulated with IL-2 (10 IU/ml) or IL-7 (1 ng/ml) for 15 min and simultaneously stained with a monoclonal antibody against CD4-PacificBlue (OKT4, BioLegend). PBMCs were immediately fixed thereafter using fixation/permeabilization buffer (True-Nuclear-kit, BioLegend) according to manufacturer’s instructions. Samples were then stained with a monoclonal antibody against pSTAT5-PE (SRBCZX, eBioScience) and measured by flow cytometry (LSRFortessa, BD). Analyses were done using FlowJo software (Miltenyi Biotech). Representative depictions of flow cytometry data are provided as part of Fig. 3b.

For the regulatory T cell assay, freshly isolated PBMC from healthy donors genotyped for *CISH* SNPs were enriched for T_REG_ using the CD4^+^CD25^+^CD127^dim/−^ Regulatory T Cell Isolation Kit II and by magnetic cell sorting (MACS) (both BD Biosciences) according to the manufacturer’s instructions. The purity of enriched T_REG_ cells was determined by flow cytometry using the following antibodies: CD4-PacificBlue (OKT4, BioLegend), CD127-A647 (HIL-7R-M21, BD Biosciences), CD25-PeCy7 (2A3, BD Biosciences). Only samples containing more than 95% enriched T_REG_ cells were included. Heterozygous CD3^+^ T cells (termed “effector T cells” (T_EFF_) throughout) were enriched using the non-contact T Lymphocyte Enrichment Kit and MACS technology (both BD Biosciences). A purity of more than 95% was confirmed by flow cytometry using monoclonal antibodies for CD3-APC (UCHT1, BD Biosciences). T_EFF_ were labeled with carboxyfluorescein succinimidyl ester (CFSE) proliferation dye (eFluor™ 450, Thermo Fisher) following the manufacturer’s instructions. T_EFF_ (1 × 10^5^) alone or in co-culture with different concentrations of T_REG_ (i.e., 2.5 × 10^4^, 5 × 10^4^, 1 × 10^5^) were then stimulated with CD3/CD28 beads (1 μl, Gibco) for 4 days in RPMI medium containing 10% human AB serum and 1% penicillin/streptomycin. CFSE dilution indicating cellular division of effector T cells was measured by flow cytometry (LSRFortessa, BD). Analyses were performed using FlowJo software (Miltenyi Biotech). Representative flow cytometry histograms are provided in Fig. 4a.

### Statistical analysis

MAFs for *CISH* SNPs were calculated according to standard methods and compared to the European (EUR) cohort from the 1000 Genomes Project [17] using Fisher’s exact test; 95% confidence intervals (CI) were calculated with the Newcombe method. The Mann-Whitney *U* test was applied to compare disease characteristics (i.e., onset age, C-peptide level, insulin dose) between haplotypes. Further, the Friedman test was used to evaluate the effect of IL-2 and IL-7 stimulation on CIS mRNA expression and pSTAT5 induction between haplotypes as well as for the effect of different T_REG_/T_EFF_ ratios on T_EFF_ proliferation. Two-tailed *p* values below 0.05 were considered statistically significant. All analyses and figure preparations were performed with GraphPad Prism (Version 7.0a. GraphPad Software) and SAS for Windows version 9.4 (SAS Institute, Cary, North Carolina, USA).

## Results

In a cohort of 260 T1D patients from the pediatric diabetes biobank (German Center for Diabetes Research, DZD), three *CISH* promoter SNPs (i.e., rs809451, rs414171, rs2239751) were analyzed. We found comparable MAFs for rs809451 (11.3%) and rs414171 (12.1%) (Table 1). This similarity was due to a strong linkage disequilibrium between these SNPs (*R*^2^ = 0.92), whereas inheritance of the rs2239751 SNP was largely independent from rs414171 (*R*^2^ = 0.06) and rs809451 (*R*^2^ = 0.002) (Fig. 1a). The rs2239751 MAF was low (0.8%) and homozygous carriers were not found (Table 1). Next, we compared MAFs of T1D patients with data from a European cohort genotyped as part of the 1000 Genomes Project (*n* = 503) (Table 1). We detected similar MAFs and no significant differences between both cohorts (Table 1).

**Fig. 2:**
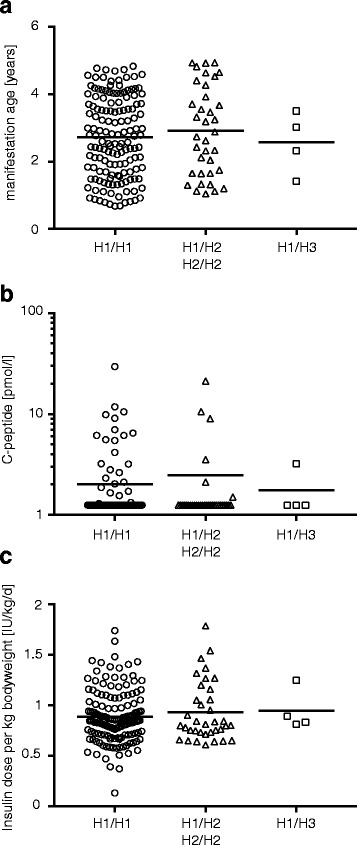
T1D patient characteristics stratified by *CISH* haplotypes. T1D patients homozygous for haplotype 1 (H1/H1, circles), H2 haplotype carriers (H1/H2 and H2/H2, triangles), and H3 haplotype carriers (H1/H3, squares) were characterized regarding age at manifestation (years) (**a**), residual C-peptide level (pmol/l) (**b**), and daily insulin dosage per kilogram body weight (IU/kg/day) (**c**). Every symbol represents the data from an individual patient, medians are indicated. No statistically significant differences were detected between study groups (Mann-Whitney *U* test, two-tailed)

Characterization of SNPs rs809451, rs414171, and rs2239751 led to the assignment of three haplotypes (H1, H2, H3, Fig. 1b). The vast majority of T1D patients had the homozygous genotype H1/H1 (*n* = 199) whereas a lower proportion was heterozygous for H1 and H2 (H1/H2, *n* = 55). Hardly any T1D patients with homozygous H2 (H2/H2, *n* = 2) or H3 (heterozygous H1/H3; *n* = 4) genotypes were identified (Fig. 2). To investigate a possible influence of haplotypes on T1D disease manifestation and severity, we compared T1D onset age, serum C-peptide levels, and insulin requirement between T1D patients with different haplotypes (Fig. 2). No differences in age at manifestation (Fig. 2a), C-peptide levels (Fig. 2b), or daily insulin requirement (Fig. 2c) were found. These results did not suggest an association of *CISH* promoter SNPs with manifestation age or severity of T1D.

**Fig. 3:**
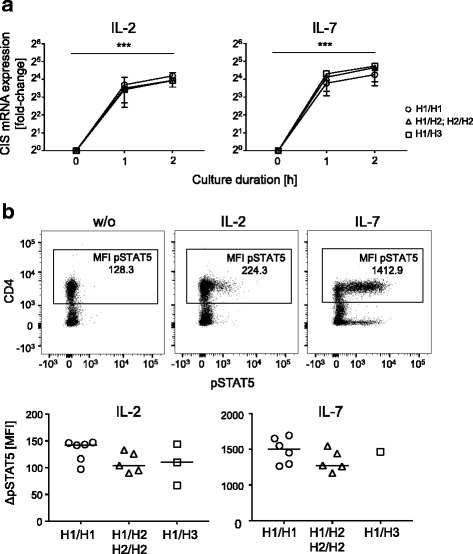
Cytokine induced CIS mRNA expression and STAT5 phosphorylation according to *CISH* haplotypes. **a** CIS mRNA expression of PBMCs from healthy adult donors with distinct haplotypes (circles: homozygous H1 carriers (H1/H1), triangles: H2 carriers (H1/H2, H2/H2), squares: H3 carriers (H1/H3)) treated with IL-2 (left graph) or IL-7 (right graph) for 1 and 2 h. CIS mRNA levels—calculated in comparison to the housekeeping gene GAPDH—are normalized against time point 0 for each donor. Median and IQR of 2^−ΔΔCT^ values are indicated. *p* value for effect of IL-2/IL-7 stimulation on CIS mRNA levels considering all genotypes (Friedman test) is indicated as *** for *p* < 0.001. For IL-2 stimulation, 5 H1/H1, 5 H2, and 3 H3 carriers were included. For IL-7 stimulation, 6 H1/H1, 5 H2, and 1 H3 carriers were analyzed. **b** IL-2 and IL-7 induced STAT5 phosphorylation of CD4^+^ T cells for different *CISH* haplotypes. Representative gating procedure on CD4^+^ T cells for pSTAT5 expression without stimulation (left chart), after stimulation with IL-2 (middle chart) and IL-7 (right chart) is shown in the upper plots. Mean fluorescence intensity values (MFI) for pSTAT5 are indicated. In the bottom charts, *∆* pSTAT5 values (MFI) (difference to pSTAT5 level in absence of IL-2 or IL-7) after stimulation with IL-2 (left panel) and IL-7 (right panel) are depicted for the different haplotype carriers. Medians are indicated. No statistically significant differences were detected between study groups (Mann-Whitney *U* test, two-tailed)

Previous studies described impaired IL-2 induced CIS expression of T cells with H2 [11, 16]. We applied IL-2 and IL-7 in vitro T cell stimulation for comparison of samples from healthy individuals assigned to the three genotypes (i.e., homozygous H1/H1, H2 carrier (H1/H2 and H2/H2), and H3 carrier (H1/H3); Fig. 3). Because of the low frequency of homozygous H2 carriers, only one homozygous H2 donor was included. In vitro stimulation with IL-2 or IL-7 for 1 and 2 h induced an increase of CIS mRNA expression (both *p* < 0.001, Fig. 3a). We detected no differences between the three genotypes 1 or 2 h after stimulation.

To determine functional effects of *CISH* haplotypes on cytokine receptor signaling, we measured IL-2 and IL-7 induced STAT5 phosphorylation for the same study groups (Fig. 3b). Both IL-2 and IL-7 increased pSTAT5 (all *p* < 0.001) without perceptible differences between the three genotypes (Fig. 3b).

**Fig. 4:**
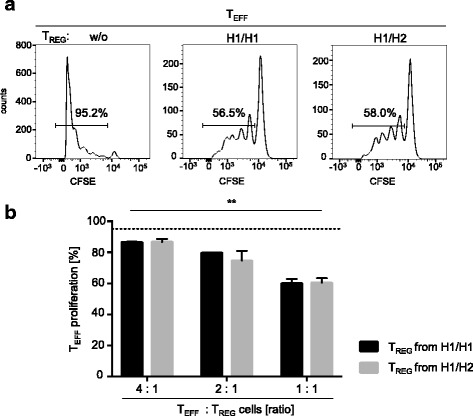
T_REG_ suppression assay. **a** Representative depiction of CFSE stainings of allogenic effector T cells (T_EFF_) incubated without regulatory T cells (T_REG_) (left chart), with T_REG_ from an H1/H1 carrier (middle chart) and T_REG_ from an H1/H2 carrier (right chart). Percentages of proliferated T_EFF_ cells are indicated. **b** Proliferation rate of T_EFF_ (%) coincubated with T_REG_ from H1/H1 carriers (black bars, *n* = 2) and from H1/H2 carriers (gray bars, *n* = 2) in different T_EFF_/T_REG_ ratios. Median and range are indicated. The dotted line indicates proliferation rate of T_EFF_ incubated without T_REG_. *p* value for the effect of T_EFF_/T_REG_ titration considering both haplotypes (Friedman test) is indicated as ** for *p* = 0.005

Finally, we compared T_REG_ from the two dominant genotypes H1/H1 and H1/H2 for their in vitro ability to limit T_EFF_ proliferation. Different proportions of T_REG_ were co-cultured with allogenic effector T cells, and effector cell proliferation was measured after 5 days (Fig. 4a). A significant reduction of T_EFF_ proliferation was detected when T_REG_ were added and the effects increased with higher T_REG_ proportions (*p* = 0.005, Fig. 4b). However, comparable T_REG_ effects were found for samples from H1/H1 and H1/H2 carriers. We concluded that no association of *CISH* SNPs with susceptibility to T1D was detectable and that haplotypes had no influence on IL-2/IL-7 signaling or T_REG_ functions.

## Discussion

This study showed that *CISH* promoter SNP rs809451, rs414171, and rs2239751 frequencies were comparable between patients with early onset of T1D and controls from the publically available 1000 Genomes database. Therefore, no evidence for an association of *CISH* promoter variants and susceptibility to develop T1D was detected. Available cohort sizes restricted the sensitivity of this study and moderate effects of *CISH* SNPs cannot be excluded. In addition, the 1000 Genomes Project cohort comprised individuals from different European regions and, therefore, effects due to ethnic differences between study groups are possible [17].

We detected no differences in T1D manifestation age between different haplotypes. However, all T1D patients included in the present study had an early onset of T1D below 5 years of age and this preselection limited differences in onset age between haplotypes. Differences in disease course are indicated by residual ß-cell activity—measured by residual C-peptide levels—and daily insulin requirements of T1D patients. We detected no differences between the haplotypes for these parameters. Thus, these results do not suggest haplotype-dependent effects on autoimmune mechanisms and disease course in T1D patients.

Previous studies suggested a role of *CISH* promoter SNPs on CIS mRNA expression [11, 16]. Whereas Khor et al. detected CIS mRNA expression differences only for the homozygous minor allele carriers (H2/H2 according to the nomenclature used here) [11], Sun et al. also detected decreased CIS mRNA for heterozygous (H1/H2) patients carrying the rs809451 minor allele [16]. Only one H2/H2 carrier was found among healthy individuals recruited and, therefore, we were not able to reproduce the results for the homozygous H2 genotype. In contrast to Sun et al. [16], we detected no differences in CIS mRNA for H1/H2 carriers as compared to H1/H1 carriers. In addition, we did not detect H1/H2 effects on IL-2 induced STAT5 phosphorylation. Differences between rs809451 and rs414171 minor allele carriers described [16] could not be reproduced in the present study because of the strong linkage disequilibrium between these SNPs.

In addition, the study by Sun et al. found differences in IL-10 serum levels of rs414171 minor allele carriers [16]. These finding suggested functional effects of *CISH* haplotypes on T_REG_, the main producers of IL-10 [21]. Our functional T_REG_ analyses of H1/H1 and H1/H2 carriers did not indicate differences in their ability to limit T_EFF_ proliferation. Since other immune cell populations (e.g., follicular T helper cells) produce IL-10 [22], described differences in IL-10 serum levels may be explained by non-T_REG_-mediated mechanisms.

Our results do not suggest a role of *CISH* SNPs in the susceptibility to T1D, whereas other reports showed an association with susceptibility to infectious diseases [11]. CIS is involved in the regulation of several cytokines including IL-7, which is crucial for naïve and memory T cell homeostasis and may promote autoreactive T cell responses in T1D [23]. Therefore, a possible role of CIS in T1D may be more complex and not based on impaired cytokine-induced CIS expression of different haplotypes. In accordance, multiple roles of CIS in different immune cell populations have been described [12, 13, 24]. Further analyses are needed to address this question.

## Conclusions

This study provides no evidence for an association of *CISH* promoter SNPs with susceptibility to T1D or severity of the disease. In contrast to previous studies that demonstrated marked effects of *CISH* SNPs on T cells, no influence of different haplotypes on CIS mRNA expression or T cell-mediated functions was found. Further studies are needed to address the question, how *CISH* variants mechanistically carry out their role during allergic, malignant, and infectious diseases.

## Additional file

Additional file 1: Figure S1. Post-hoc power calculations and confidence intervals for MAF differences. (PPTX 99 kb)

## Abbreviations

CIS: Cytokine-inducible SH2-containing protein
IL-2R: Interleukin-2 receptor
MAFs: Minor allele frequencies
PBMCs: Peripheral blood mononuclear cells
SNP: Single nucleotide polymorphism
T1D: Type 1 diabetes
T_EFF_: Effector T cells
T_REG_: Regulatory T cells
