# Supplementary material for: CISH promoter polymorphism effects on T cell cytokine receptor signaling and type 1 diabetes susceptibility
Source: Mol Cell Pediatr. 2018 Feb 6;5:2. doi: 10.1186/s40348-018-0080-7 (PMC5801137; doi:10.1186/s40348-018-0080-7)
Supplement: Additional file 1: Figure S1. — Post-hoc power calculations and confidence intervals for MAF differences. (PPTX 99 kb) [file 40348_2018_80_MOESM1_ESM.pptx]

## Slide 1
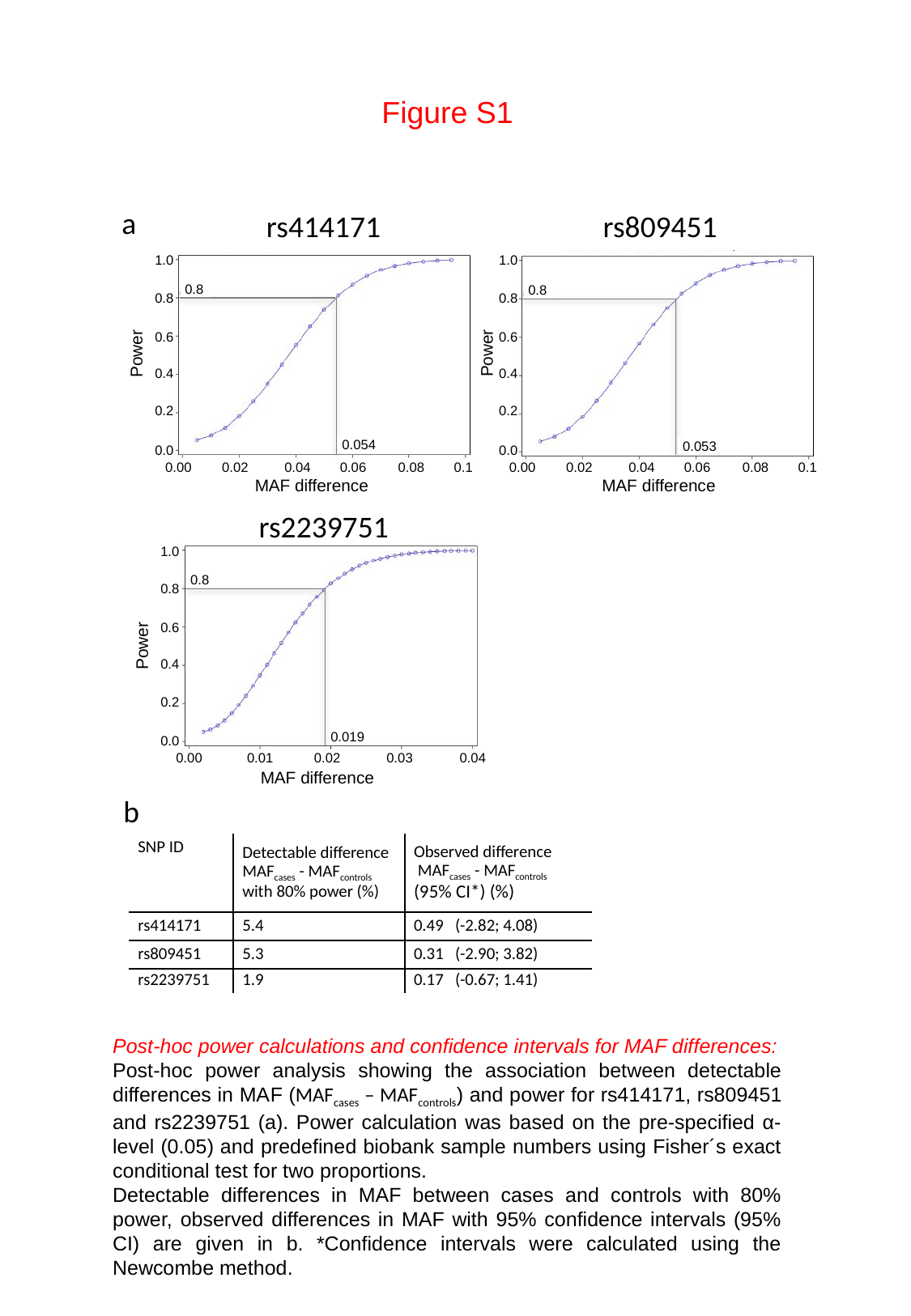

Figure S1
a
rs414171
rs809451
1.0
0.8
0.6
0.4
0.2
0.0
0.02
0.04
0.06
0.08
0.1
0.00
1.0
0.8
0.6
0.4
0.2
0.0
0.02
0.04
0.06
0.08
0.1
0.00
0.8
0.8
Power
Power
0.054
0.053
MAF difference
MAF difference
rs2239751
1.0
0.8
0.6
0.4
0.2
0.0
0.01
0.02
0.03
0.04
0.00
0.8
Power
0.019
MAF difference
b
| SNP ID | Detectable difference MAFcases - MAFcontrols with 80% power (%) | Observed difference MAFcases - MAFcontrols (95% CI\*) (%) |
| --- | --- | --- |
| rs414171 | 5.4 | 0.49 (-2.82; 4.08) |
| rs809451 | 5.3 | 0.31 (-2.90; 3.82) |
| rs2239751 | 1.9 | 0.17 (-0.67; 1.41) |
Post-hoc power calculations and confidence intervals for MAF differences:
Post-hoc power analysis showing the association between detectable differences in MAF (MAFcases – MAFcontrols) and power for rs414171, rs809451 and rs2239751 (a). Power calculation was based on the pre-specified α-level (0.05) and predefined biobank sample numbers using Fisher´s exact conditional test for two proportions.
Detectable differences in MAF between cases and controls with 80% power, observed differences in MAF with 95% confidence intervals (95% CI) are given in b. *Confidence intervals were calculated using the Newcombe method.
